# Anaplastic Lymphoma Kinase-Positive Large B-Cell Lymphoma: An Underrecognized Aggressive Lymphoma

**DOI:** 10.1155/2012/529572

**Published:** 2012-02-26

**Authors:** Elizabeth A. Morgan, Alessandra F. Nascimento

**Affiliations:** Department of Pathology, Brigham and Women's Hospital, Boston, MA 02115, USA

## Abstract

Anaplastic lymphoma kinase-(ALK-) positive large B-cell lymphoma (ALK+ LBCL) is a rare, aggressive tumor characterized by an immunoblastic or plasmablastic morphologic appearance, expression of ALK, CD138, CD45, EMA, and often IgA by immunohistochemistry, and characteristic chromosomal translocations or rearrangements involving the *ALK* locus. The morphologic and immunophenotypic overlap of this tumor with other hematologic and nonhematologic malignancies may result in misdiagnosis. The tumor has been identified in both pediatric and adult populations and demonstrates a male predominance. Presentation is most often nodal, particularly cervical. No association with immunocompromise or geographic location has been recognized. The most common gene rearrangement is between *clathrin* and *ALK* (t(2;17)(p23;q23)), resulting in the CLTC-ALK chimeric protein, although other fusions have been described. Response to conventional chemotherapy is poor. The recent introduction of the small molecule ALK inhibitor, crizotinib, may provide a potential new therapeutic option for patients with this disease.

## 1. Introduction

Anaplastic lymphoma kinase-(ALK-) positive large B-cell lymphoma (ALK+ LBCL) was first described by Delsol and colleagues in 1997 [1] and is now listed as a distinct entity in the 2008 WHO Classification of Tumours of Haematopoietic and Lymphoid Tissues [2]. ALK+ LBCL is an aggressive tumor with a poor response to conventional therapies. Although it appears to be very rare, it may in fact be underrecognized due to its morphologic and immunophenotypic overlap with other hematologic and nonhematologic entities. Awareness of this diagnosis, particularly in a new era of ALK inhibitor therapies, is necessary for hematopathologists as well as general surgical pathologists.

## 2. Clinical Features and Epidemiology

Fewer than 100 cases of ALK+ LBCL have been described in the literature (reviewed in [3–6], subsequent cases reported in [7–13]). The neoplasm has been diagnosed in both pediatric and adult age groups, ranging in age from 9 to 85 years old, with a median age of 37 to 44.5 years, and with a male predominance of 3–5 : 1. The proportion of patients under age 18 has been found to comprise approximately 15–20% of the total. One confirmed case occurring in a patient with the human immunodeficiency virus (HIV) has been reported [12]. In general, patients do not appear to be immunocompromised. Furthermore, patients with this tumor do not appear to be restricted geographically; cases have been reported from Europe, the United States, and Asia. Our institution recently diagnosed ALK+ LBCL on a biopsy sample from a 29-year-old man living in Rwanda [S. Rodig, personal communication].

ALK+ LBCL most commonly presents in lymph nodes, particularly cervical, although extranodal involvement has been reported in a wide variety of sites, including the GI tract, epidural space, ovaries, skeleton, nasopharyngeal or nasal area, tongue, brain, and liver. Bone marrow and splenic involvement have also been described. The majority of patients present with advanced stage disease. No tumors have been positive for the Epstein-Barr virus (EBV) by in situ hybridization or for human herpes virus-8 (HHV8) by immunohistochemistry [5, 6].

## 3. Diagnosis

**Figure 1:**
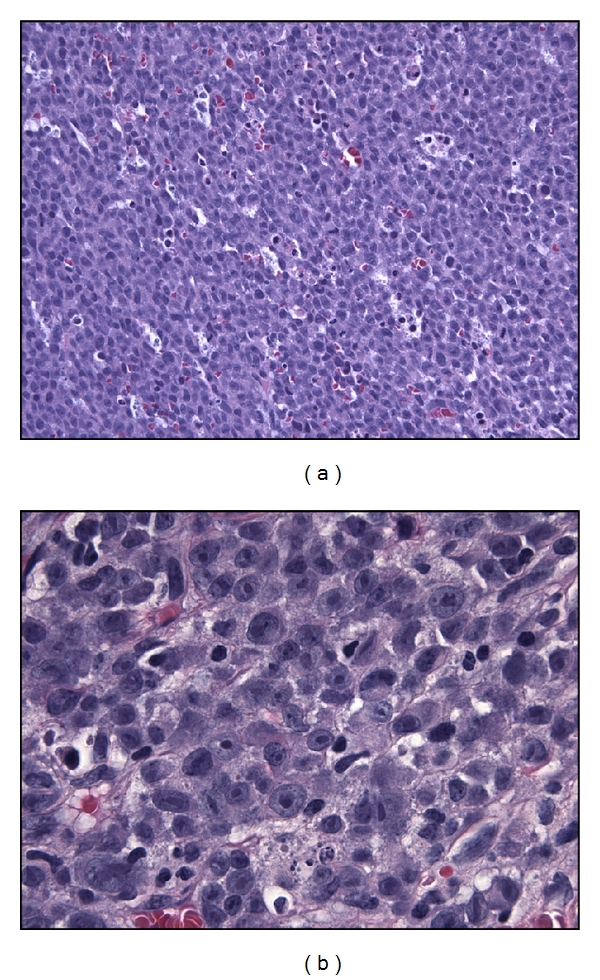
ALK+ LBCL morphology. (a) The tumor grows diffusely with a sheet-like architecture (400x, H&E). (b) The tumor cells are intermediate to large sized with round nuclei, dispersed chromatin, centrally placed nucleoli and moderate amounts of amphophilic cytoplasm (1000x, H&E).

**Figure 2:**
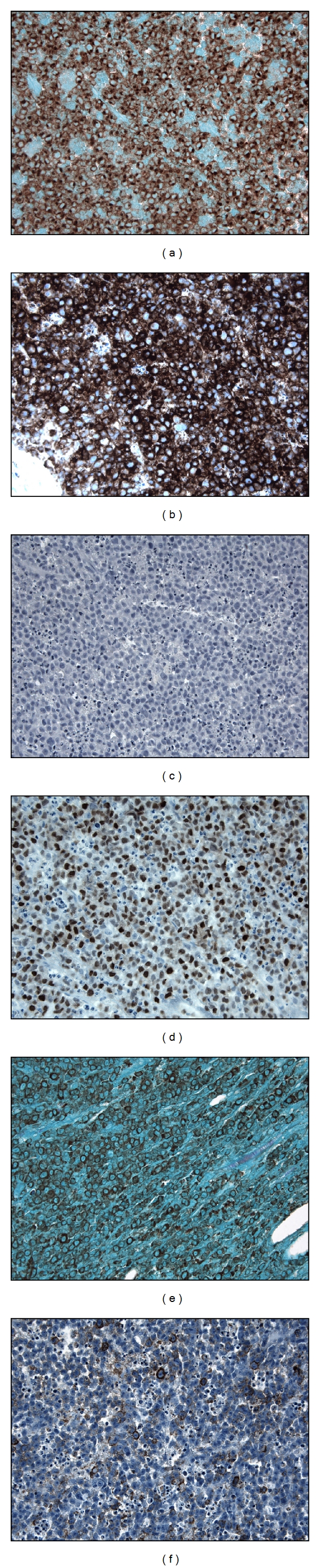
ALK+ LBCL immunohistochemical profile. All tumors are positive for ALK (a) and CD138 (b). CD20 (c) is almost always negative, but MUM-1 (d) is frequently positive. IgA is often expressed (e). EMA can show focal to diffuse positivity (f). All microphotographs are 400x.

The diagnosis of ALK+ LBCL requires synthesis of the morphologic features and immunohistochemical findings and is aided by cytogenetic data when available. The neoplastic cells are intermediate to large sized, and immunoblastic- or plasmablastic-appearing with round nuclei, dispersed chromatin, a single, prominent, central nucleolus and moderate amounts of eosinophilic to amphophilic cytoplasm (Figure 1). The tumor grows in sheets in nodal and extranodal sites, and, within the lymph node, often demonstrates sinusoidal invasion. The tumor cells are almost always negative for CD20 and CD79a but are always positive for CD138 and CD38, consistent with a postgerminal center phenotype (Figure 2). ALK stains 100% of tumors, and its staining pattern may be suggestive of the underlying cytogenetic abnormality (see Genetics and Pathogenesis). CD45, EMA, and MUM-1 are also frequently positive, and CD4 staining may be seen. The tumors often express IgA with monotypic light chain restriction. Other markers, including keratins, have infrequently been reported.

Due to the morphologic and immunophenotypic overlap with other hematologic and nonhematologic malignancies, this neoplasm may be misdiagnosed. Indeed, several cases in the literature were initially classified as poorly differentiated or anaplastic carcinoma [6], ALK-positive anaplastic large cell lymphoma (ALK+ ALCL) [14], or extramedullary plasmacytoma [5, 10]. Further, the plasmablastic morphologic features overlap with other aggressive large B-cell lymphomas with plasmablastic differentiation. It is possible that the small number of cases in the literature may represent underreporting due to failure of recognition of the entity. Immunohistochemical studies can greatly assist in the interpretation of these tumors (Table 1).

## 4. Genetics and Pathogenesis

Anaplastic lymphoma kinase (ALK) is a tyrosine kinase of the insulin receptor superfamily. In all cases of ALK+ LBCL in which cytogenetic or molecular attempts were successful, chromosomal translocation or rearrangement involving the *ALK* locus has been identified by karyotype, FISH, and/or RT-PCR. Although it was initially suggested that a full-length ALK protein was expressed by these tumors, it is now clear that these tumors commonly express the product of a fusion gene that incorporates the 3′ portion of *ALK* encoding the tyrosine kinase domain.

Several genetic partners fused to *ALK* have been described. The most common is *clathrin (CLTC-ALK)*, the product of t(2;17)(p23;q23) (first described in [15–17]). Interestingly, this translocation has been described in less than 1% of cases of ALK+ ALCL [2] and has also been identified in inflammatory myofibroblastic tumors (IMT) [18]. In both ALK+ LBCL and ALK+ ALCL, immunohistochemical staining with anti-ALK antibodies gives a granular cytoplasmic pattern in neoplastic cells because clathrin encodes for a coated vesicle protein involved in intracellular transport. Less commonly described in ALK+ LBCL is the nucleophosmin-ALK (NPM-ALK) fusion protein, resulting from t(2;5)(p23;q35) [9, 19, 20]. In contrast, NPM-ALK is the most common chimeric protein in ALK+ ALCL [2]. In both ALK+ LBCL and ALK+ ALCL bearing this chimeric protein, immunohistochemical staining with ALK gives a nuclear and cytoplasmic staining pattern. As in ALK+ ALCL, the distinct ALK staining patterns may act as a surrogate for the underlying gene rearrangement in ALK+ LBCL.

As awareness of this rare lymphoma increases, investigators are using more sensitive and directed techniques to confirm the presence of ALK rearrangements in tumors that immunophenotypically are compatible with the diagnosis. Van Roosbroeck and colleagues utilized sophisticated technique including metaphase FISH, RT-PCR, and 5′ RACE PCR to identify a cryptic *SEC31A-ALK* fusion generated by insertion of the 5′ end of *SEC31A* (4q21) upstream of the 3′ end of *ALK* [9]. This had previously only been described in one case of IMT [21] and was subsequently confirmed in a second case of ALK+ LBCL by Bedwell and colleagues [8]. In all three cases, the *SEC31A-ALK* fusion was the result of complex rearrangements rather than a single reciprocal translocation event (which would not be possible due to the opposite transcriptional orientation of *ALK* and *SEC31A*); this likely accounts for the rarity of both its occurrence and its detection. As pointed out by Van Roosbroeck et al., Stachurski and colleagues' report of an ALK+ LBCL with complex karyotype and cryptic insertion of 3′-*ALK* gene sequences into chromosome 4q22-24 may, potentially, represent a *SEC31A-ALK* fusion [22]. In all cases, ALK immunohistochemical staining was granular cytoplasmic.

In addition to these reproducible genetic events, additional case reports have demonstrated 5′*ALK* gene deletion [23]; duplication of the *ALK* gene region/additional copy of chromosome 2 [24]; complex karyotype with two independent *ALK* translocations: t(X;2)(q21;p23) and t(2;12)(p23;q24.1) showing granular cytoplasmic ALK staining [12]; *SQSTM1-ALK*, generated from t(2;5)(p23.1;q35.3), showing diffuse cytoplasmic ALK staining with ill-demarcated spots [13].

Several experimental models support the role of *ALK* fusion genes in disease pathogenesis. Expression of a *SEC31A-ALK* construct in the interleukin-3-(IL3-) dependent cell line Ba/F3 permitted growth factor-independent growth upon cessation of IL3 administration [9]. Addition of an ALK inhibitor resulted in decreased ALK tyrosine phosphorylation and decreased phosphorylation of downstream effectors ERK1/2 and STAT3, and, less robustly, AKT and STAT5 [9]. STAT3, but not STAT5, has been found to be highly hyperphosphorylated on tyrosine 705 in two cases of ALK+ LBCL with the *CLTC-ALK* fusion protein [7]. Injection of 3T3 cells expressing *SQSTM1-ALK *into nude mice produced subcutaneous tumors [13]. A *CLTC-ALK*-positive DLBCL cell line, generated from a patient with systemic relapsed disease, formed subcutaneous tumors in NOD-SCID mice, which morphologically and immunophenotypically recapitulated the ALK+ LBCL phenotype, including the granular cytoplasmic ALK staining pattern [14]. As in ALK+ ALCL, one could hypothesize that ALK chimeric proteins allow for constitutive activation of the ALK tyrosine kinase independent of ligand binding, resulting in unchecked activation of downstream effectors.

## 5. Outcome and Treatment Strategies

As opposed to ALK+ ALCL, which has an overall 5-year survival rate approaching 80% [2], ALK+ LBCL has a dismal prognosis and poor response to conventional therapy regimens. Laurent and colleagues retrospectively analyzed clinical outcome data in 31 patients diagnosed with ALK+ LBCL [6]. All patients with documented therapy (*n* = 30) received cyclophosphamide, doxorubicin, vincristine, and prednisone (CHOP) or CHOP-derived chemotherapy; 11 additionally received radiation therapy, and 3 underwent subsequent autologous stem cell transplantation. The 5-year survival rate was 25% with a median survival of 12 months in advanced stage disease. Overall survival was significantly shorter for patients with stage III or IV disease as defined by the Ann Arbor staging system compared to those who presented with stage I or II disease.

Another review of 46 published cases and 4 new cases of ALK+ LBCL analyzed outcomes in 41 cases with available treatment data [5]. Twelve of 32 patients treated with chemotherapy received CHOP; of these, 6 patients relapsed and 4 patients died of progressive lymphoma. Of all treated patients, eighteen (44%) had relapsed/refractory disease, and 7 of 8 patients who underwent salvage hematopoietic stem cell transplantation (4 autologous, 1 allogeneic, 3 unspecified) died between 3 and 44 months after diagnosis. Notably, a subsequently published case report described relapse and death within 100 days of front-line autologous transplantation after treatment with CHOP and radiation therapy [10]. Overall, Beltran and colleagues documented that 56% of reported patients died, most commonly (90% of cases) due to progressive lymphoma, with an overall survival time of 24 months. Similarly to Laurent and colleagues, they found that the strongest correlative factor to survival was clinical stage at presentation, calculating an 18-month median survival in patients with advanced disease versus not reached in early-stage presentations [5].

While a systematic, prospective study of treatment regimens would be difficult in this rare disease, it is apparent from the reported literature that ALK+ LBCL is an aggressive disease with poor response to conventional therapies. More recently, interest has turned to the potential use of a new class of drugs, namely, ALK inhibitors. In addition to ALK+ LBCL, mutations or gene rearrangements leading to growth-factor-independent ALK activation have been implicated in the oncogenesis of several neoplasms including ALK+ ALCL [25], 50% of IMTs [26, 27], a subset of sporadic and familial neuroblastoma [28, 29], and 5% of nonsmall cell lung cancer (NSCLC) [30, 31]. The use of a small molecular ALK inhibitor is an attractive possibility in these diseases, and the potential is garnering excitement in the oncology field.

Recently, and in a remarkably short time since the first description of the ALK rearrangement in NSCLC, the Food and Drug Administration approved the use of crizotinib (Xalkori Capsules, Pfizer, Inc.), the small-molecule dual inhibitor of the c-Met and ALK receptor tyrosine kinases, for the treatment of patients with locally advanced or metastatic NSCLC with an ALK rearrangement [30]. Crizotinib has also been tried on a case-by-case basis in other tumors. Butrynski and colleagues recently reported a sustained partial response in a patient with an ALK-translocated IMT, while no response was observed in a patient with an ALK-non-rearranged IMT [32]. Gambacorti-Passerini and colleagues administered crizotinib to two patients with relapsed ALK+ ALCL; clinical improvement was seen within one week for both patients, and complete response was sustained at six months and five months, respectively [33].

There is some experimental evidence to suggest that ALK inhibitors may be efficacious in the treatment of ALK+ LBCL. Upon identifying a *SEC31A-ALK* fusion in a patient with ALK+ LBCL, Van Roosbroeck and colleagues demonstrated that the selective ALK inhibitor NVP-TAE684 (TAE-684) could inhibit the growth of a Ba/F3 cell line expressing the *SEC31A-ALK* construct in a dose-dependent manner [9, 34]. Cerchietti and colleagues established a cell line expressing CLTC-ALK, the most common fusion protein identified in ALK+ LBCL, and demonstrated that TAE-684 could inhibit cell growth *in vitro* and could regress murine tumor xenografts *in vivo* [14]. To date, no reports of the use of crizotinib in patients with ALK+ LBCL have appeared in the literature. However, at least one Phase 1B clinical trial, studying the safety and clinical activity of crizotinib in tumors with genetic events involving the *ALK* gene locus, is currently recruiting patients (NCT01121588), and reference was made to a patient enrolling in an inhibitor trial in a recent case report [12].

## 6. Conclusion

ALK+ LBCL is a rare, aggressive B-cell lymphoma with characteristic morphologic, immunophenotypic and cytogenetic/molecular findings. Awareness of this entity is important in both the hematopathology and general surgical pathology fields. Although response to conventional therapy has been poor, the possibility of a targeted therapy provides an intriguing option for patients with this disease.

## Figures and Tables

**Table 1:** Differential diagnosis of ALK+ LBCL and predominant staining patterns with several antibodies. This table summarizes the predominant staining patterns for these entities, with the understanding that exceptional cases have been reported. Typically, ALK+ LBCL demonstrates a postgerminal center phenotype and is negative for CD30. Rare cases expressing CD20, CD79a, or CD30 have been reported [5].

	ALK+ LBCL	ALK+ ALCL	DLBCL, NOS	Poorly differentiated carcinoma	Extramedullary plasmacytoma
CD45	+	+	+	−	
CD20	−	−	+		−
CD79a	−		+		+
MUM-1	+		+/−		
CD138	+	−	−	+/−	+
ALK	+	+	−	−	−
CD30	−	+	+/−		
EMA	+	+		+/−	
Keratin	+/−			+/−	
EBER	−	−			
